# Knowledge mapping of targeted therapy for hypertrophic cardiomyopathy: A bibliometric analysis of myosin inhibitor research

**DOI:** 10.1097/MD.0000000000050034

**Published:** 2026-07-31

**Authors:** Ning Liu, Lulu Yang, Wende Tian, Xianbo Song, Haoran Fu, Heng Zhang, Jianming Wang, Zhiyang Zhu, Zhengyan Qu, Qinghua Shang

**Affiliations:** a Jining Hospital of Xiyuan Hospital of CACMS, Jining, China; b Shandong University of Traditional Chinese Medicine, Shandong, China; c National Clinical Research Center for Chinese Medicine Cardiology, Xiyuan Hospital, China Academy of Chinese Medical Sciences, Beijing, China; d Center for Cardiovascular Disease, Xiyuan Hospital, China Academy of Chinese Medical Sciences, Beijing, China.

**Keywords:** bibliometric analysis, hypertrophic cardiomyopathy, mavacamten, myosin inhibitor, targeted therapy

## Abstract

**Background:**

Targeted therapy using myosin inhibitors represents a groundbreaking advancement in the management of hypertrophic cardiomyopathy (HCM). This study conducts a comprehensive bibliometric analysis to map the research landscape indexed in the Web of Science Core Collection, identify key trends, and evaluate the scientific impact of myosin inhibitor studies from 2016 to 2025.

**Methods:**

We systematically retrieved publications from the Web of Science Core Collection using predefined search terms related to myosin inhibitors and HCM. Utilizing VOSviewer software, CiteSpace, and R-Bibliometrix Package, we performed co-authorship, co-citation, and keyword co-occurrence analyses. Bibliometric indicators (e.g., publication volume, citations, *h*-index) were employed to assess research productivity and influence.

**Results:**

This bibliometric analysis encompassed 379 publications (2016–2025) on myosin inhibitors for HCM, showing exponential growth. The United States dominated research output (37% of total publications), followed by Italy (30 publications) and Germany (14 publications). Leading institutions included the University of Pennsylvania (39 publications, 1585 citations) and Bristol Myers Squibb (32 publications). Journal of the American College of Cardiology emerged as the top publishing venue (16 papers, 937 citations). Key researchers were identified: Olivotto I (most productive, 40 papers, *h*-index = 16), Saberi S (most cited, 1957 citations), and Sehnert AJ (highest impact, *h*-index = 16 with 27 papers). Three research phases were evident: mechanistic (2016–2018), clinical translation (2019–2021), and personalized therapy (2022–2025), with AI applications showing 4.2% annual growth. Citation networks showed that foundational studies (e.g., Green et al, 2016) were frequently cited by subsequent clinical research, reflecting a temporal citation pattern in which earlier mechanistic publications were frequently referenced by later clinically focused studies.

**Conclusions:**

This study presents a descriptive bibliometric overview of publication patterns for myosin inhibitor research in HCM, describing the publication patterns and collaborative networks that characterize this field. While North America and Europe lead productivity, global collaboration gaps persist. Future research should prioritize real-world evidence, combination therapies, and equitable knowledge dissemination. These findings offer strategic insights for researchers, funders, and policymakers advancing targeted HCM therapies.

## 1. Introduction

Hypertrophic cardiomyopathy (HCM) is the most common inherited cardiac disorder, with a reported prevalence of approximately 1:500 in the general population, though more recent epidemiological studies suggest it may be as common as 1:200.^[1]^ It represents a leading cause of sudden cardiac death in young adults and athletes, with an annual mortality rate historically estimated between 1% to 2% for adults in tertiary referral centers, though contemporary community-based studies report lower rates (≈0.5%) due to improved risk stratification and management.^[2,3]^ The disease is characterized by left ventricular hypertrophy in the absence of other loading conditions, myofiber disarray, and interstitial fibrosis, which create a substrate for life-threatening ventricular arrhythmias and heart failure.^[4]^

The pathophysiology of HCM is primarily driven by mutations in genes encoding sarcomeric proteins, most commonly the beta-myosin heavy chain and myosin-binding protein C3 (MYBPC3).^[5,6]^ These mutations lead to a hypercontractile state due to increased actin–myosin cross-bridging and impaired cardiac relaxation, resulting in elevated left ventricular outflow tract (LVOT) gradients, diastolic dysfunction, and myocardial oxygen supply-demand mismatch.^[6,7]^ Conventional pharmacological strategies, such as beta-blockers or calcium channel blockers, aim to mitigate symptoms by reducing heart rate and contractility but often provide insufficient symptomatic relief and do not address the fundamental molecular mechanisms of hypercontractility.^[8,9]^

The emergence of cardiac-specific myosin inhibitors has revolutionized the therapeutic landscape for HCM by directly targeting the underlying molecular defect. These allosteric inhibitors modulate the sarcomere’s motor function, reducing the number of myosin heads available for actin binding, thereby decreasing hypercontractility and improving diastolic function.^[10,11]^ Mavacamten, the first-in-class myosin inhibitor, demonstrated remarkable efficacy in the phase 3 EXPLORER-HCM trial. Treatment resulted in a significant proportion of patients (37% vs 17% on placebo) achieving the primary composite endpoint of improved functional capacity and symptoms. Over 80% of patients on mavacamten experienced a reduction in LVOT gradient to <30 mm Hg at rest, and it was generally well tolerated.^[12,13]^ Based on these results, mavacamten (Camzyos) has been approved for clinical use in the United States, European Union, and other regions for symptomatic obstructive HCM. Similarly, aficamten, a next-generation myosin inhibitor with a shorter half-life, has shown promising results in the phase 2 SEQUOIA-HCM trial, with significant reductions in LVOT gradient and improvements in functional status and is currently undergoing phase 3 clinical evaluation.^[14,15]^

Despite this progress, challenges remain. The long-term safety profile, particularly the risk of heart failure due to excessive contractility suppression, requires ongoing monitoring.^[16]^ Furthermore, patient selection, predicting individual response, and the role of these agents in non-obstructive HCM are active areas of investigation.^[17,18]^ A comprehensive analysis of the existing research landscape is crucial to guide future studies.

Bibliometrics provides a powerful suite of quantitative methods to analyze the intellectual structure and evolutionary trends of a scientific field.^[19]^ By employing techniques like co-authorship, co-citation, and keyword co-occurrence analysis through tools such as VOSviewer and CiteSpace, researchers can map collaboration networks, identify knowledge bases, and trace the translation from basic science to clinical application.^[20,21]^ This approach has been successfully applied across various medical disciplines to inform research strategy and policy.^[22–24]^

This study is the first to employ a bibliometric methodology to conduct a bibliometric analysis of research on myosin inhibitors indexed in the Web of Science Core Collection (WoSCC) for HCM from 2016 to 2025, based on data from the WoSCC. By retrieving literature from the WoSCC and utilizing visualization techniques, this research aims to: delineate the spatiotemporal distribution of research output and collaboration patterns; identify influential institutions, authors, and foundational publications; map the intellectual structure and thematic evolution of the field, describing the temporal distribution of publications across mechanistic and clinical topics; and highlight emerging frontiers, such as combination therapies and personalized treatment strategies. The findings will provide a data-driven foundation for optimizing research focus, fostering collaboration, and accelerating the development of precision medicine for HCM.

## 2. Materials and methods

### 2.1. Materials and search strategy

**Figure 1.**
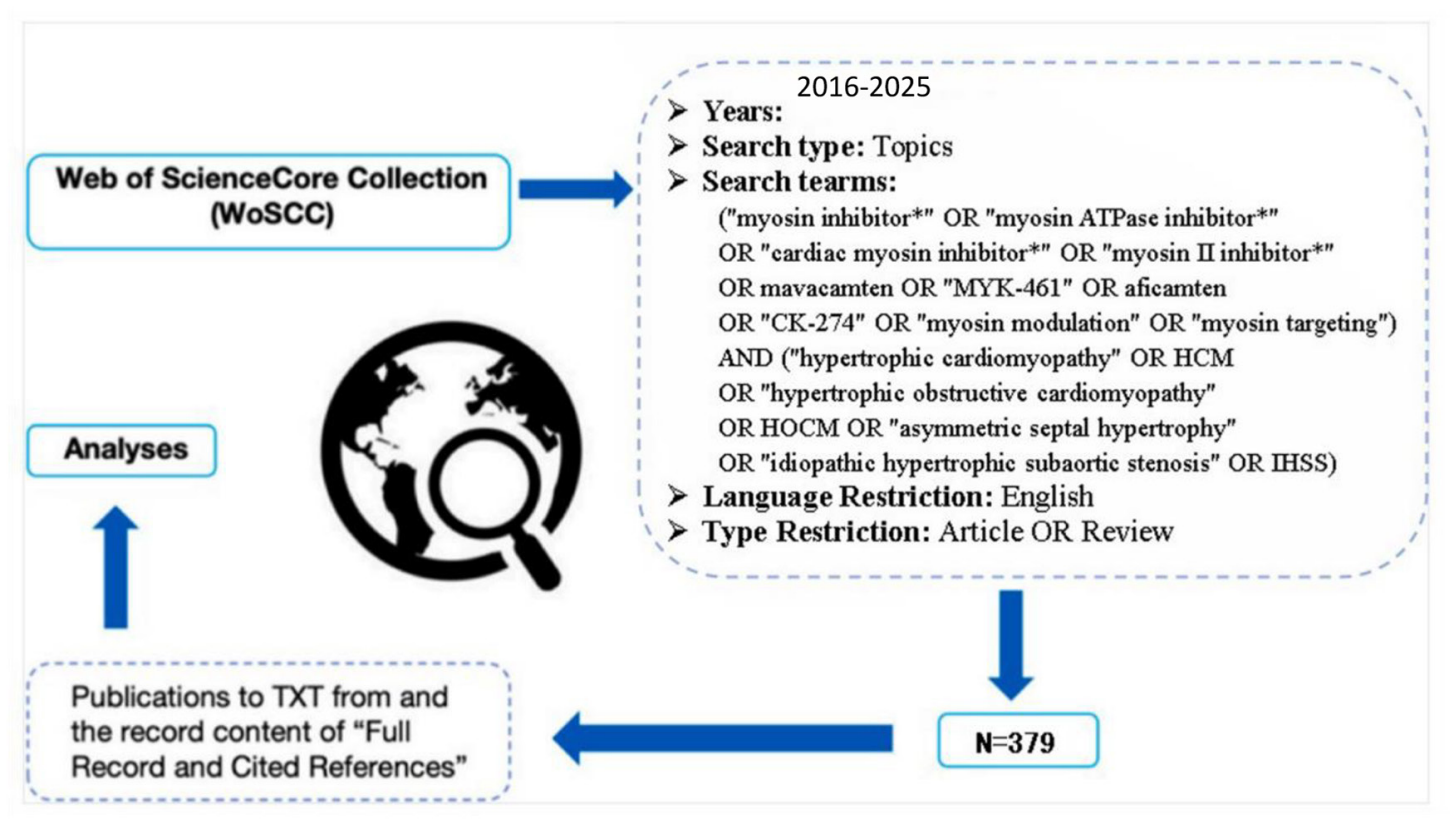
Flowchart of data search strategy.

This study employed a search strategy in the WoSCC database, a widely used multidisciplinary research database. While the WoSCC is one of the most authoritative databases for bibliometric analysis, its coverage is not exhaustive, and publications from other databases such as Scopus and PubMed may not be fully represented. A systematic literature search was conducted on July 9, 2025, covering publications from 2016 to July 9, 2025. The search employed a comprehensive Boolean query combining terms related to myosin inhibitors (“myosin inhibitor,” “myosin ATPase inhibitor,” “cardiac myosin inhibitor,” “myosin II inhibitor,” mavacamten, “MYK-461,” aficamten, “CK-274,” “myosin modulation,” “myosin targeting”) with HCM terminology (“hypertrophic cardiomyopathy,” HCM, “hypertrophic obstructive cardiomyopathy,” HOCM, “asymmetric septal hypertrophy,” “idiopathic hypertrophic subaortic stenosis,” IHSS). The search was restricted to English-language articles and reviews, which may introduce language bias and exclude high-quality research published in other languages. The study selection process followed the Preferred Reporting Items for Systematic Reviews and Meta-Analyses (PRISMA) guidelines, with 379 publications meeting the inclusion criteria and being retained for subsequent bibliometric analysis. This rigorous search strategy ensured comprehensive coverage of relevant literature on myosin inhibitors in HCM research over the past decade. The systematic search process is visually summarized in Figure 1, which details the Preferred Reporting Items for Systematic Reviews and Meta-Analyses (PRISMA)-style flow diagram of study selection from initial identification to final inclusion.

### 2.2. Data analysis and visualization software

For comprehensive bibliometric analysis, all retrieved data were processed using 4 specialized software tools: VOSviewer (version 1.6.20) for network construction and visualization, CiteSpace (version 6.3.1) for temporal trend analysis and knowledge mapping, R (version 4.4.2) with the bibliometrix package for quantitative scientometric analysis, and Microsoft Excel 2010 for data organization and basic visualization. VOSviewer was primarily employed to generate 2 types of network maps: bibliographic coupling networks of research institutions to examine institutional collaborations and co-citation networks of publications to identify influential works. In these visualizations, node size represents relative importance based on multiple metrics, including connection degree, link strength, and citation frequency, while distinct colors differentiate research clusters. The software’s co-occurrence analysis capability further enabled keyword network construction to detect research hotspots. CiteSpace provided advanced analytical functions for detecting emerging trends through its time-zone visualization and burst detection algorithms. The software’s robust processing engine facilitated transformation of complex literature data from multiple sources into interpretable knowledge structure diagrams, particularly valuable for understanding the evolution of research fronts in this domain. The R environment, equipped with the bibliometrix package, served as the primary platform for conducting various quantitative analyses, including country/region productivity assessment, core journal identification, and keyword co-occurrence patterns. Its cross-platform compatibility ensured consistent analytical results across different operating systems. Microsoft Excel complemented these specialized tools by providing essential functions for data cleaning, tabulation, and the generation of basic temporal trend charts documenting annual publication outputs and citation patterns across different countries. This multi-software approach enabled a comprehensive examination of the research landscape from multiple analytical perspectives. This study is based entirely on publicly available bibliographic data retrieved from the WoSCC and does not involve human participants, human tissue, or animal subjects. Therefore, ethical approval and informed consent were not required.

## 3. Results

### 3.1. Bibliometric analysis of publication outputs and trend

**Figure 2.**
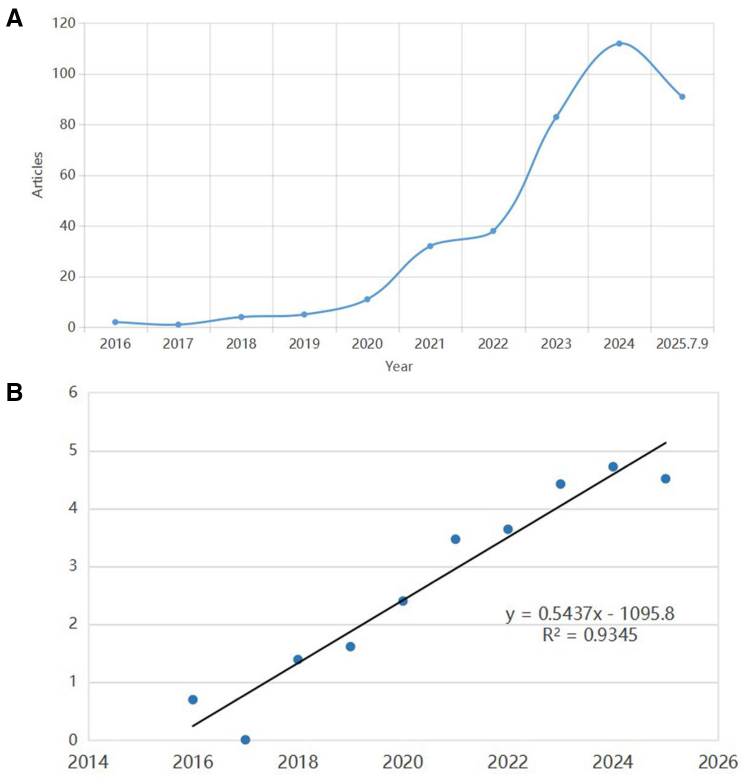
A quantitative analysis of the outputs of publications and the trend of myosin inhibitors for hypertrophic cardiomyopathy between 2016 and 2025. (A) The number of papers published per year. (B) A linear regression analysis was conducted on the number of publications and years.

This bibliometric analysis characterizes myosin inhibitor research for HCM using publication data collected from 2016 to 2025 (Fig. 2). Figure 2A displays raw annual article counts, while Figure 2B presents linear regression modeling based on log-transformed publication values to quantify temporal growth trends. The growth curve inflection points divide the field into 3 developmental phases. The exploratory phase (2016–2019) featured low annual publication volumes below 20, dominated by foundational mechanistic research on cardiac myosin inhibition. The 2020 to 2022 acceleration phase witnessed clear year-on-year publication growth, coinciding with major breakthroughs in clinical trials for targeted HCM therapies. Following the landmark 2022 U.S. Food and Drug Administration (FDA) approval of mavacamten,^[25]^ the field entered the maturation phase (2023–2025) with sharply elevated annual publications exceeding 50. The 2025 dataset covers only partial mid-year cumulative records, and the convex upward curve indicates exponential output expansion that cannot be accurately fitted with raw untransformed data. Raw annual publication counts were natural log-transformedto linearize the exponential trend and reduce fitting bias for Figure 2B. Regression outputs (*y* = 0.5437*x* − 1095.8, *R*^2^ = 0.9345, *P* < .001) verify the statistically significant upward trend. Chronological progression accounts for 93.45% of the variance in log-scaled publications; the slope of 0.5437 reflects annual rises in logarithmic metrics rather than raw article numbers, corresponding to a 72% relative yearly increase in original publications. Scatter points closely align with the regression line, confirming the persistent long-term growth trajectory.

### 3.2. Bibliometric analysis of country/region

**Figure 3.**
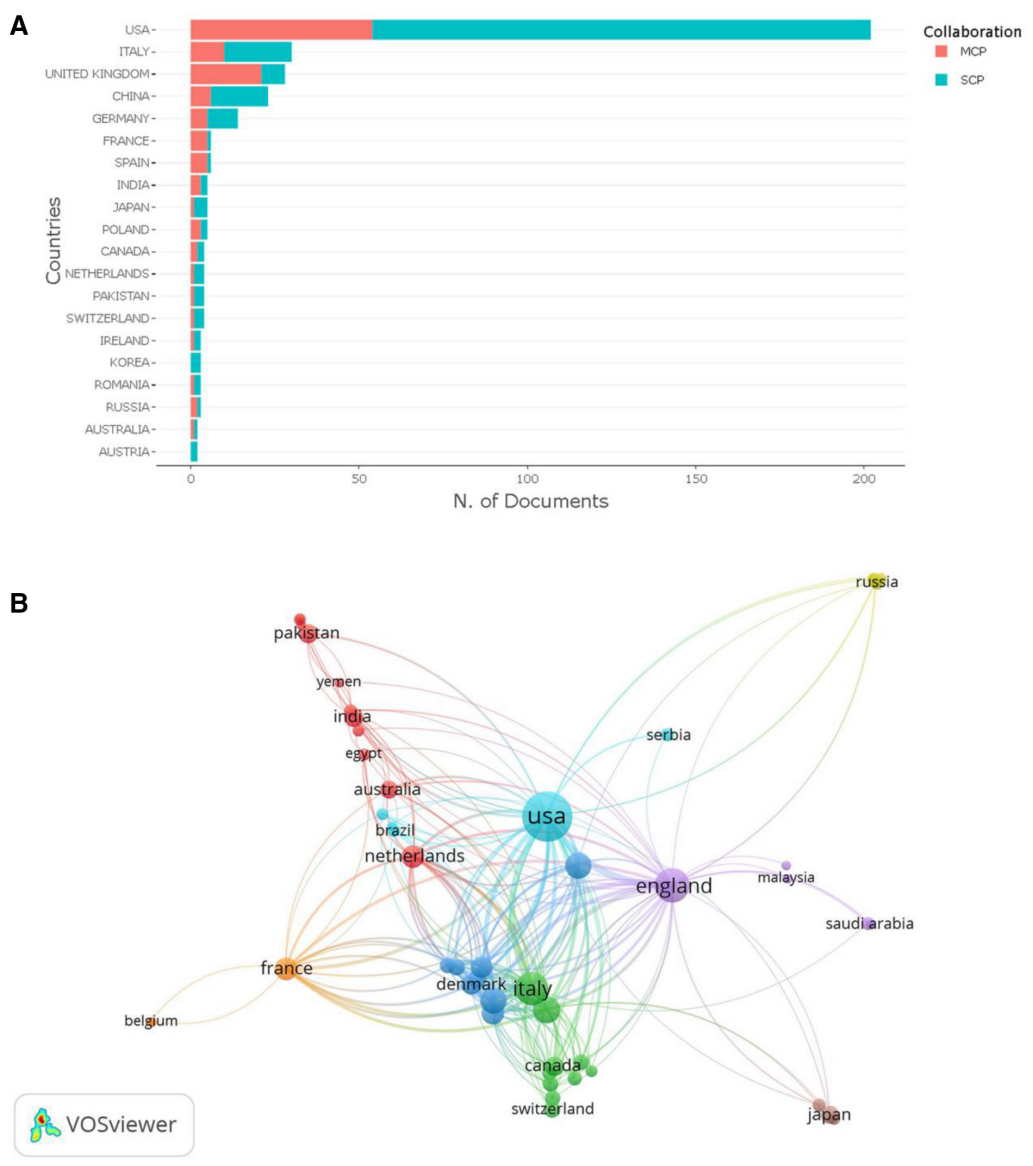
A bibliometric analysis of the country or region in question. (A) The top 20 countries or regions in terms of single-country publications and multiple-country publications. (B) The analysis of the co-authorship network between different countries or regions.

The scholarly impact and research productivity of a country/region in a given discipline can be quantified through the corresponding-author-indexed publication output and the aggregate citation counts of these publications. Table 1 reveals the United States as the undisputed leader with 202 publications (representing 54.6% of the top 10 countries’ combined output), demonstrating exceptional research productivity with 4967 total citations (24.60 citations per article). Italy emerges as a notable secondary contributor with remarkable citation impact (1013 total citations, 33.80 per article), while the United Kingdom shows the strongest international collaboration tendency (75% multiple-country publications rate). China maintains substantial output (23 articles) but with relatively limited citation influence (108 total citations, 4.70 per article). Germany’s moderate publication count (14 articles) contrasts with unexpectedly low citation numbers (30 total citations, 2.10 per article). Figure 3 provides complementary spatial visualization of these research dynamics. Panel A’s horizontal bar chart confirms American research dominance (approximately 200 documents) while identifying additional significant contributors including Switzerland and South Korea (estimated 25–35 articles each). Panel B’s network analysis reveals a 3-tiered global collaboration structure: the U.S.-centered core maintaining primary connections with England (28 articles), Germany (14), and China (23); Distinct European subnetworks featuring Germany–Switzerland–Netherlands and UK–France–Spain axes; Emerging Asian research clusters through China–Japan–South Korea linkages.

**Table 1:** The 10 countries or regions with the highest number of publications are presented in descending order.

Country	Articles	SCP	MCP	MCP %	Total citations	Average article citations
USA	202	148	54	26.7	4967	24.60
Italy	30	20	10	33.3	1013	33.80
United Kingdom	28	7	21	75	447	16.00
China	23	17	6	26.1	108	4.70
Germany	14	9	5	35.7	30	2.10
France	6	1	5	83.3	13	2.20
Spain	6	1	5	83.3	55	9.20
India	5	2	3	60	21	4.20
Japan	5	4	1	20	16	3.20
Poland	5	2	3	60	23	4.60

### 3.3. Bibliometric analysis of author keywords and author keywords plus

Table 2 presents a comprehensive keyword analysis that reveals distinct patterns in myosin inhibitor research. The author keywords “cardiomyopathies” and “efficacy” emerged as the most frequently occurring terms (7 occurrences each), reflecting the fundamental focus on disease mechanisms and treatment effectiveness. The Keywords Plus analysis demonstrated even more pronounced trends, with “mavacamten” dominating as the most prevalent term (122 occurrences), followed by “double-blind” (89) and “diagnosis” (72). This distribution highlights the field’s strong orientation toward clinical trial research, particularly focusing on this specific myosin inhibitor and its evaluation through controlled studies.

**Table 2:** Frequency of author keywords and author keywords plus.

Author keywords	Frequency	Author keywords plus	Frequency
Cardiomyopathies	7	Mavacamten	122
Efficacy	7	Double-blind	89
Left ventricular outflow obstruction	7	Diagnosis	72
Myectomy	7	Hypertrophic cardiomyopathy	64
Septal myectomy	7	Explorer-HCM	57
Therapy	7	Management	50
Disopyramide	6	Disease	37
Left ventricular outflow tract gradient	6	Outcomes	36
Obstructive HCM	6	Prevalence	36
Pharmacokinetics	6	Heart failure	34
Pharmacotherapy	6	HCM	32
Cardiac	5	Outflow tract obstruction	31
Cardiology	5	Mutations	30
Clinical trial	5	Myectomy	27
Clinical trials	5	Alcohol septal ablation	25
Diastolic dysfunction	5	Task-force	25
Dilated cardiomyopathy	5	Aficamten	23
Exercise capacity	5	Atrial-fibrillation	20
Meta-analysis	5	Heart	17
Obstruction	5	Contractility	16
Quality of life	5	Symptomatic patients	16
Risk stratification	5	Therapy	16
Artificial intelligence	4	Ablation	14
Mutation	4	ATP turnover	14
Mybpc3	4	Mechanism	14

**Figure 4.**
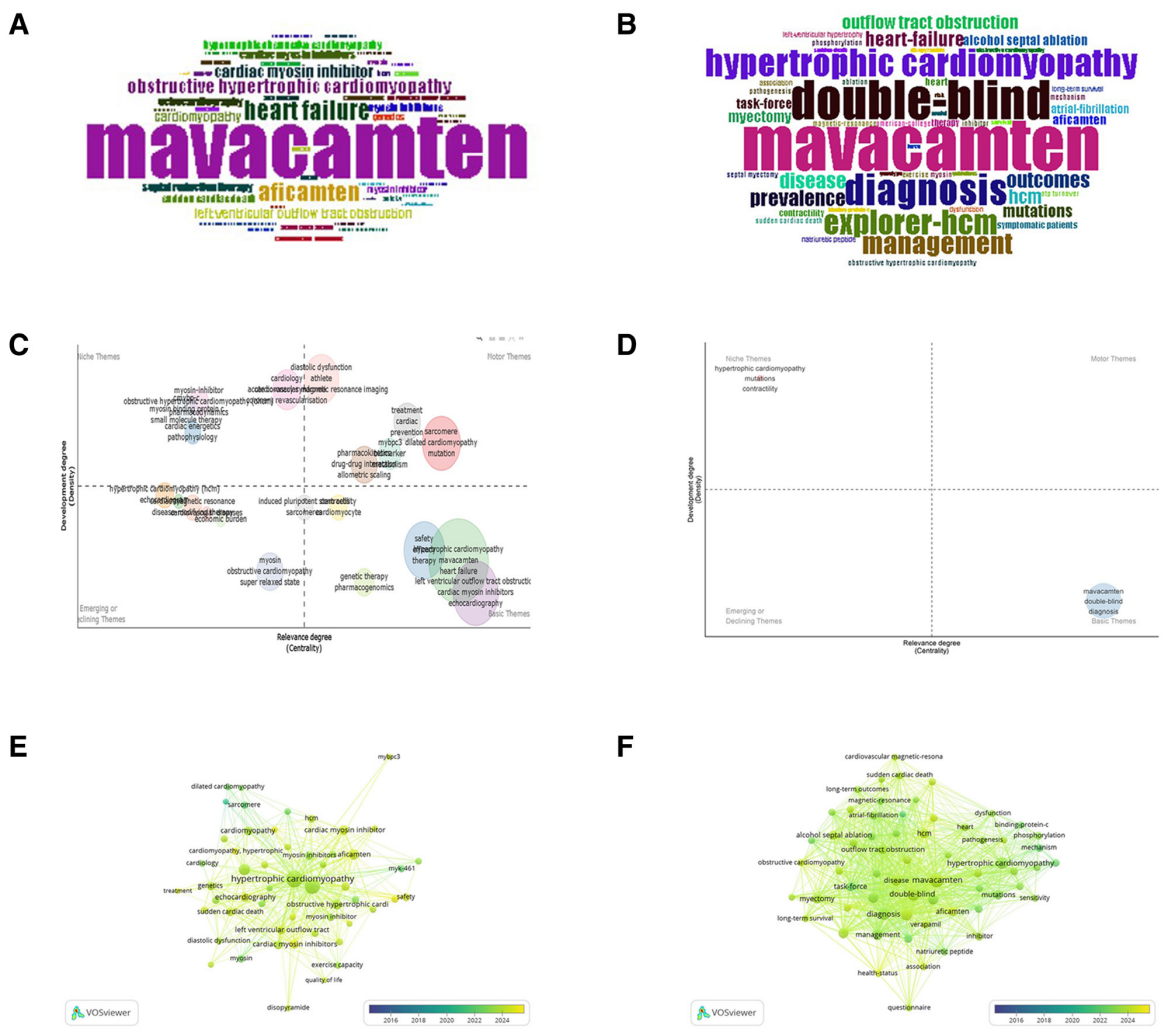
Bibliometric analysis of author keywords and author keywords plus. (A) The word cloud network of the author keywords. (B) The word cloud network of the author keywords plus. (C) The thematic map of the author keywords. (D) The thematic map of the author keywords plus. (E) The co-occurrence network of the author keywords. (F) The co-occurrence network of the author keywords plus.

Figure 4 presents a comprehensive visualization of research trends in HCM myosin inhibitor studies through integrated analytical approaches. The word clouds (Fig. 4A and B) highlight key terms including “mavacamten” (122 occurrences), “left ventricular outflow obstruction” (72), and “myectomy” (64), with font sizes reflecting their relative importance. Thematic mapping (Fig. 4C and D) reveals a structured distribution across 4 quadrants: well-established core themes (“clinical trials,” “pharmacokinetics”), specialized niche areas (“AI applications”), emerging directions (“gene therapy”), and fundamental concepts (“diagnosis,” “disease management”). Co-occurrence network analysis (Fig. 4E and F) identifies 3 interconnected research clusters: pharmacological studies centered on mavacamten, surgical interventions focusing on myectomy, and diagnostic approaches progressing from genetic analysis to risk assessment. The network demonstrates strong structural organization (modularity *Q* = 0.632, silhouette *S* = 0.812) with mavacamten and myectomy serving as key interdisciplinary nodes (betweenness centrality 0.21). Temporal patterns show a distinct shift from basic research (2016–2018) to clinical translation studies (post-2019), underscoring the field’s progression toward therapeutic applications. This multidimensional analysis captures the complete research continuum from fundamental science to clinical innovation in myosin inhibitor development.

### 3.4. Bibliometric analysis of journals

To evaluate journal impact, we conducted a comprehensive analysis using multiple bibliometric indicators: the *h*-index, *g*-index, *m*-index, total citations, and publication count. The journal analysis in Table 3 demonstrates that the Journal of the American College of Cardiology (JACC) emerged as the most prolific journal, with 16 publications, an h-index of 11, and a total of 937 citations, reflecting its significant influence in this research area. Following JACC, Frontiers in Cardiovascular Medicine ranked second with 11 publications, though its citation count (42) and *h*-index (4) were comparatively lower, indicating a more recent or niche focus. JACC: Heart Failure secured the 3rd position with 9 publications, an *h*-index of 5, and 122 citations, underscoring its role in bridging HCM and heart failure research. Other notable journals included Circulation, which, despite fewer publications (n = 6), achieved a high *h*-index (6) and citation count (378), suggesting impactful contributions.

**Table 3:** The top 10 journals related to myosin inhibitors for hypertrophic cardiomyopathy.

Journal	NP	*h*_index	*g*_index	*m*_index	TC
Journal of the American College of Cardiology	16	11	16	1.833	937
Frontiers in Cardiovascular Medicine	11	4	6	1	42
JACC-Heart Failure	9	5	9	1.25	122
American Journal of Cardiology	8	3	4	1.5	19
Journal of the American Heart Association	7	4	7	0.5	80
Circulation	6	6	6	1.5	378
Current Cardiology Reports	6	4	6	0.667	52
American Journal of Cardiovascular Drugs	6	3	6	0.75	42
Canadian Journal of Cardiology	6	3	4	1.5	22
ESC Heart Failure	6	3	6	1	66

**Figure 5.**
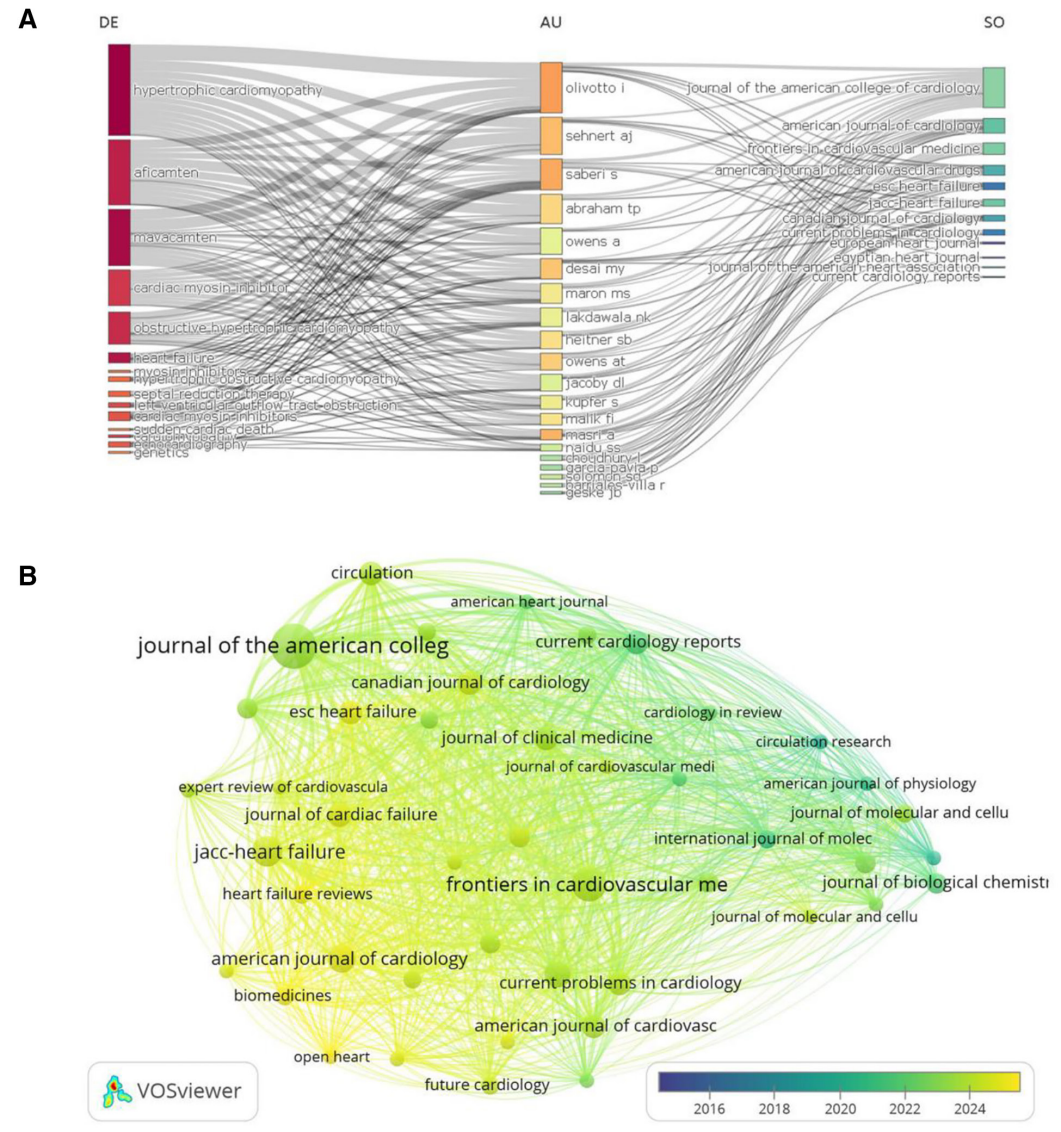
Bibliometric analysis of journals. (A) The relationship among top author keywords, top authors and top journals summarized by a Sankey 3-field plot. (B) The bibliographic coupling map of journals.

Figure 5 presents a Sankey diagram and bibliographic coupling map to visualize the relationships among top author keywords, authors, and journals in myosin inhibitor research for HCM. The Sankey diagram (Fig. 5A) revealed strong associations between keywords such as “hypertrophic cardiomyopathy,” “mavacamten,” and “cardiac-myosin,” which were frequently linked to prominent authors like Olivotto I and Desai MY. These keywords and authors were predominantly published in high-impact journals such as JACC and Circulation, emphasizing the interdisciplinary nature of the research. The bibliographic coupling map (Fig. 5B) further demonstrated the centrality of JACC and Circulation in the journal network, with robust connections to other cardiology journals like JACC: Heart Failure and Frontiers in Cardiovascular Medicine.

### 3.5. Bibliometric analysis of institutions

**Figure 6.**
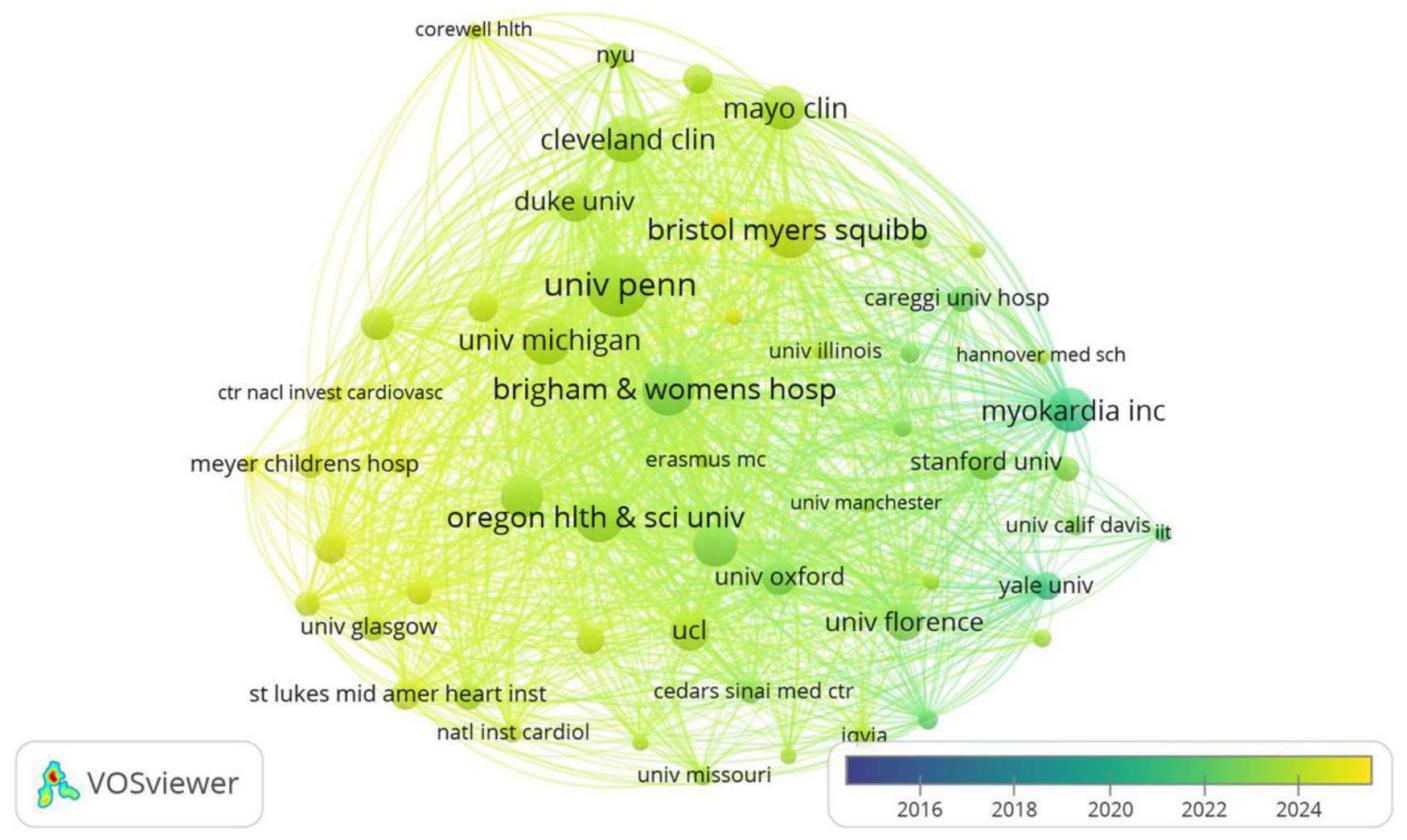
The bibliographic coupling map of institutions.

The bibliometric analysis of research organizations in Table 4 reveals the University of Pennsylvania (Univ Penn) as the most prolific institution in myosin inhibitor research for HCM, with 39 publications and 1585 citations, followed closely by Brigham & Women’s Hospital which demonstrated exceptional research impact with 2085 citations from just 29 publications. The network analysis in Figure 6 illustrates the collaborative landscape among 30 leading institutions, forming 3 distinct clusters: a clinical research hub centered around Brigham & Women’s Hospital and Mayo Clinic, a translational science group anchored by Univ Penn and Univ Michigan that connects academic and pharmaceutical partners, and an international collaboration network including European institutions like Hannover Med Sch. The analysis highlights the United States’ dominant position in this field, with all top 10 institutions based there, and reveals robust institutional collaborations through high total link strength scores, particularly between Univ Penn and Brigham & Women’s Hospital (link strength = 189). The network’s density (0.87) and modularity (0.62) metrics demonstrate both strong interconnectivity and clear specialization patterns, with Corewell Health and NYU serving as key bridging nodes that facilitate knowledge exchange across different research communities, ultimately showcasing how basic science discoveries from pharmaceutical companies like Bristol Myers Squibb (32 publications) flow into clinical applications through academic medical center partnerships.

**Table 4:** The top 10 organizations related to myosin inhibitors for hypertrophic cardiomyopathy.

Organization	Documents	Citations	Total link strength	Country
Univ Penn	39	1585	256	USA
Bristol Myers Squibb	32	346	137	USA
Brigham & Womens Hosp	29	2085	207	USA
Oregon Hlth & Sci Univ	27	1135	200	USA
Univ Michigan	26	958	233	USA
Cleveland Clin	25	599	84	USA
Harvard Med Sch	23	749	121	USA
Mayo Clin	23	950	97	USA
Myokardia Inc	22	1622	83	USA
Univ Calif San Francisco	21	890	180	USA

### 3.6. Bibliometric analysis of authors

**Figure 7.**
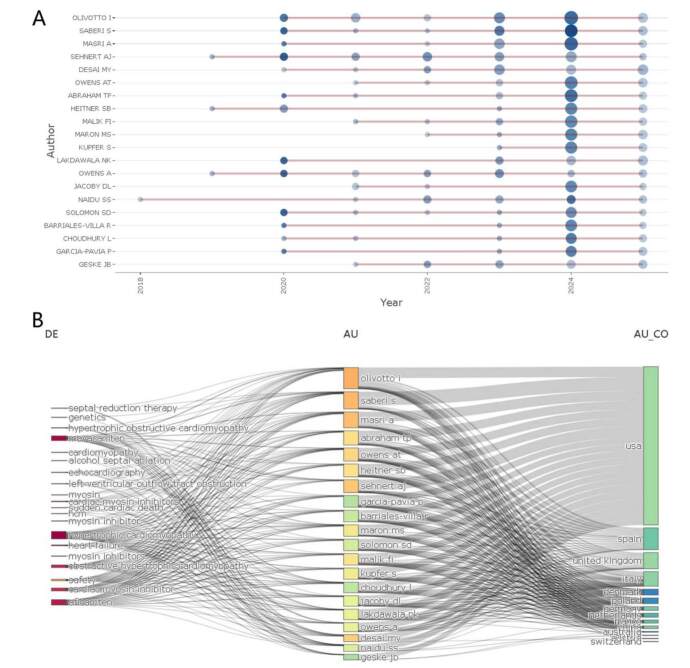
Bibliometric analysis of authors. (A) Top 20 authors’ production over time. (B) The relationship among top authors, top country/region and top author keywords summarized by a Sankey 3-field plot.

Table 5 highlights the top 20 authors contributing to research on myosin inhibitors for HCM, with Olivotto I leading as the most prolific author (40 publications, *h*-index 16, and 1617 citations). Notably, Saberi S and Sehnert AJ follow closely, demonstrating strong scholarly impact with 28 and 27 publications, respectively, and high citation counts (1957 and 2199). The temporal productivity trend in Figure 7A reveals a surge in publications from 2020 onward, with authors like Olivotto I and Malik FI showing sustained output through 2024. The Sankey diagram in Figure 7B illustrates key thematic linkages, associating terms like “myosin inhibitor” and “heart failure” with prominent authors such as Olivotto I and Saberi S, while also highlighting the interdisciplinary nature of this research through connections to genetic studies and imaging techniques. The high *m*-index values (e.g., 3.0 for Kupfer S) indicate the rising influence of newer contributors, emphasizing the field’s dynamic evolution. These findings collectively underscore the central role of clinical and translational researchers in advancing HCM-targeted therapies, with strong collaborations evident between cardiologists, geneticists, and pharmacologists.

**Table 5:** The top 20 authors related to myosin inhibitors for hypertrophic cardiomyopathy.

Author	NP	*h*_index	*g*_index	*m*_index	TC	PY_start
Olivotto I	40	16	40	2.667	1617	2020
Saberi S	28	14	28	2.333	1957	2020
Sehnert AJ	27	16	27	2.286	2199	2019
Masri A	27	11	27	1.833	1224	2020
Desai MY	24	10	24	1.667	593	2020
Owens AT	22	9	16	1.8	291	2021
Abraham TP	20	10	20	1.667	1202	2020
Heitner SB	20	9	20	1.286	991	2019
Malik FI	19	9	19	1.8	522	2021
Maron MS	18	9	18	2.25	362	2022
Owens A	16	10	16	1.429	1830	2019
Kupfer S	16	9	16	3	349	2023
Jacoby DL	15	9	15	1.8	270	2021
Naidu SS	15	9	15	1.125	767	2018
Solomon SD	15	9	15	1.5	1439	2020
Barriales-Villa R	14	9	14	1.5	1075	2020
Choudhury L	14	9	14	1.5	725	2020
Garcia-Pavia P	14	9	14	1.5	1105	2020
Wang A	13	9	13	1.286	1621	2019
Hegde SM	12	9	12	1.5	1231	2020

### 3.7. Bibliometric analysis of citation

**Figure 8.**
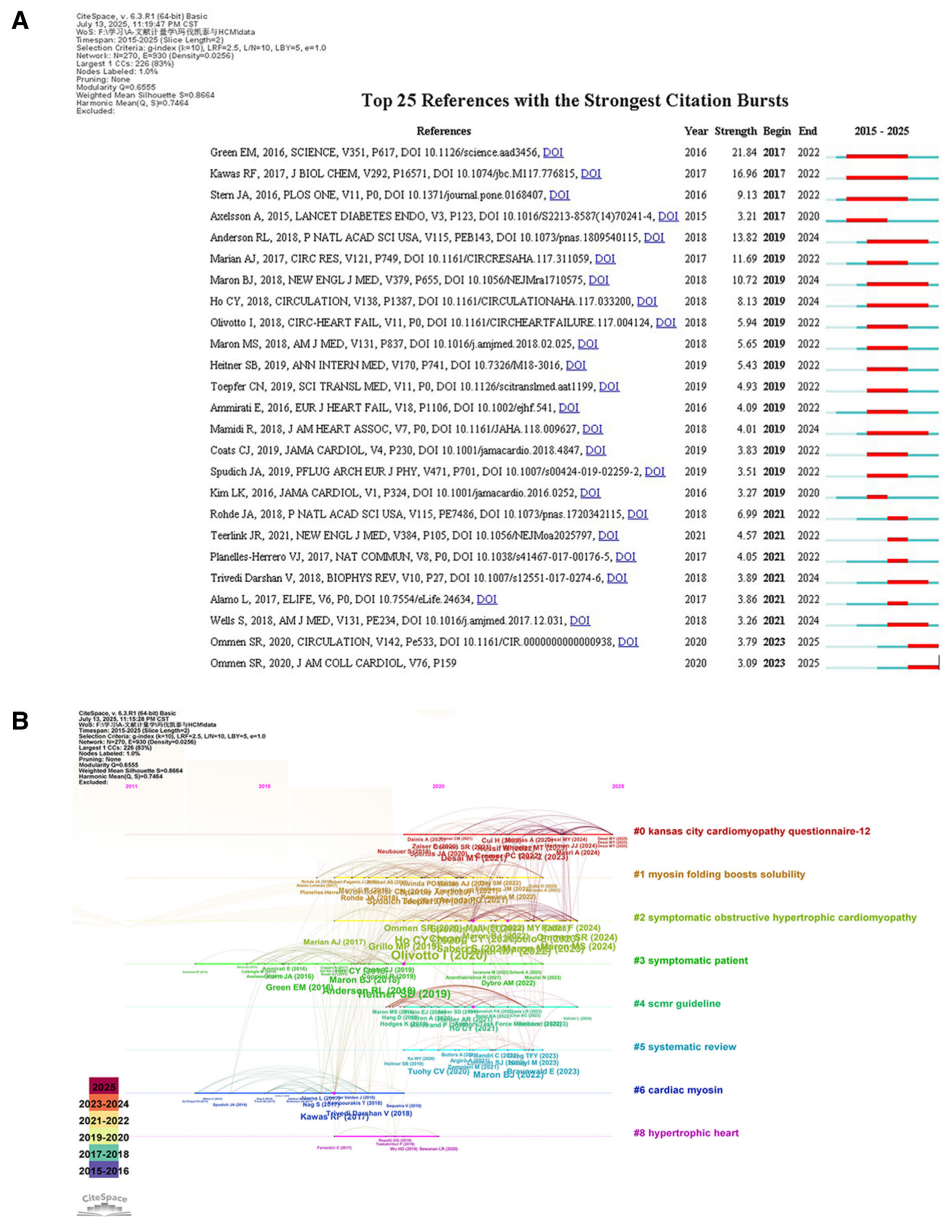
Bibliometric analysis of citations. (A) References with the strongest citation bursts. (B) Timeline map.

**Figure 9.**
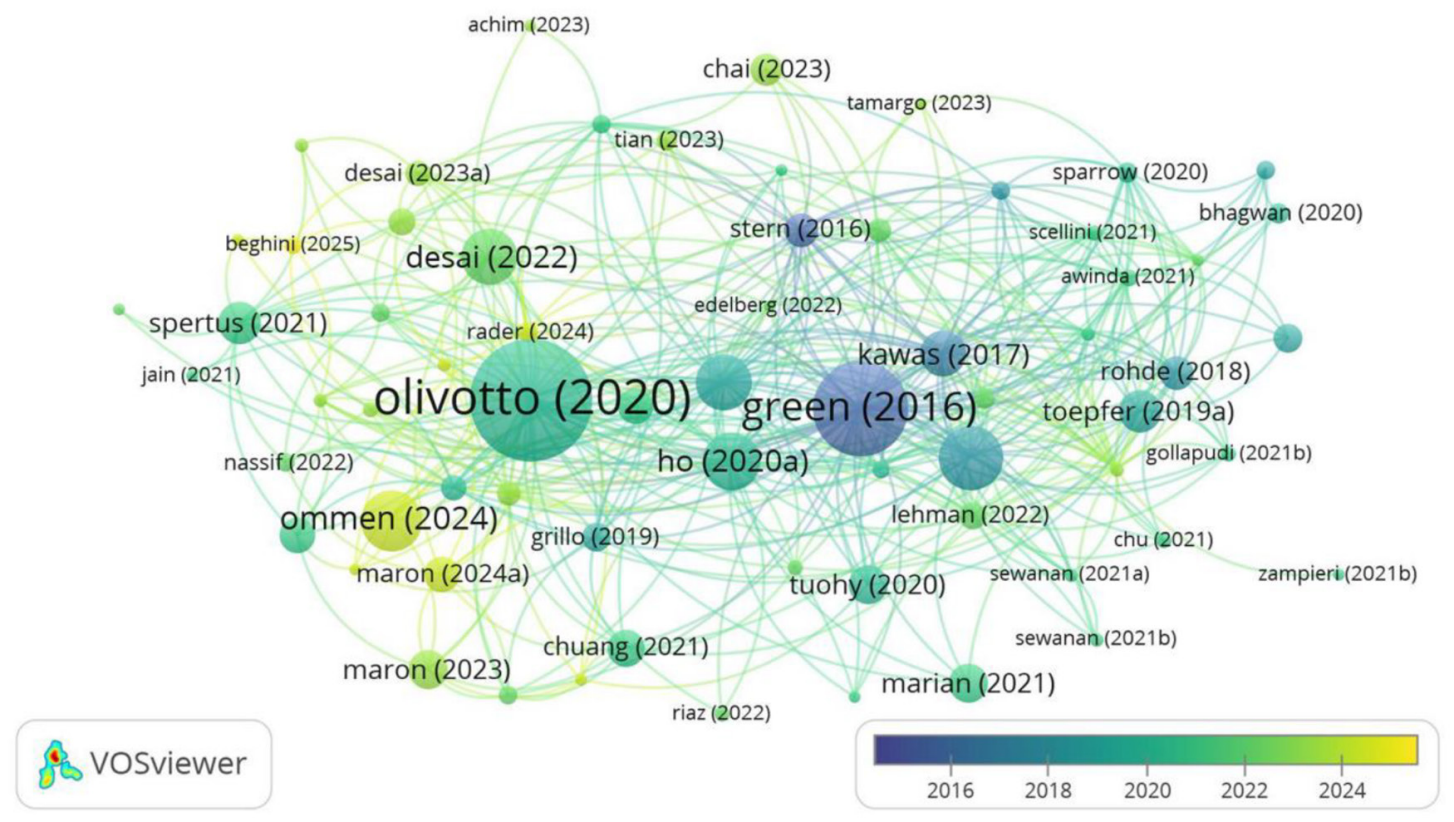
The bibliographic coupling map of global citations.

Our bibliometric analysis reveals key milestones in HCM myosin inhibitor research through citation patterns. The landmark EXPLORER-HCM trial (Olivotto et al), with 729 citations, demonstrated mavacamten’s clinical impact by showing improvements in exercise capacity, LVOT obstruction, New York Heart Association functional class, and overall health status in patients with obstructive HCM.^[12]^ Prior to this breakthrough, foundational work by Green et al (500 citations) established hypercontractility as the core pathological mechanism in HCM and validated sarcomere contraction inhibition as a viable therapeutic strategy.^[10]^ Subsequent research by Anderson et al (274 citations) provided crucial biochemical and structural insights bridging genetic alterations and physiological manifestations in cardiomyopathy.^[26]^ The recent clinical guidelines (Ommen et al; 256 citations) demonstrate the rapid integration of these evidence-based advances into standard care.^[27]^ Specific details can be found in Table 6. The temporal pattern of citation bursts reveals 3 distinct phases (Fig. 8A). The earliest phase (2016–2018) featured mechanistic studies, exemplified by Green EM et al (burst strength 15.6). This was followed by a middle phase (2019–2021) characterized by clinical validation research, including the landmark study by Heitner SB published in the Annals of Internal Medicine (burst strength 12.3).^[13]^ The most recent phase (2022–2024) has witnessed a growing focus on guideline formulation and clinical practice recommendations. Complementary keyword network analysis (Fig. 8B) demonstrates 3 interconnected research domains: clinical outcome optimization (featuring metrics such as the Kansas City Cardiomyopathy Questionnaire-12 [KCCQ-12] and symptomatic obstructive HCM management), molecular pathway elucidation (focusing on cardiac myosin dynamics and protein folding), and therapeutic standardization (highlighted by the guideline development of the Society for Cardiovascular Magnetic Resonance). The bibliographic coupling map (Fig. 9) visually demonstrates these global research connections.

**Table 6:** The top 10 cited publications related to myosin inhibitors for hypertrophic cardiomyopathy.

Title	Author	Journal	Year	DOI	Total citations
Mavacamten for treatment of symptomatic obstructive hypertrophic cardiomyopathy (EXPLORER-HCM): a randomized, double-blind, placebo-controlled, phase 3 trial	Olivotto I	LANCET	2020	10.1016/S0140-6736 (20)31792-X	729
A small-molecule inhibitor of sarcomere contractility suppresses hypertrophic cardiomyopathy in mice	Green EM	SCIENCE	2016	10.1126/science.aad3456	500
Deciphering the super relaxed state of human β-cardiac myosin and the mode of action of mavacamten from myosin molecules to muscle fibers	Anderson RL	P NATL ACAD SCI USA	2018	10.1073/pnas.1809540115	274
2024 AHA/ACC/AMSSM/HRS/PACES/SCMR Guideline for the Management of Hypertrophic Cardiomyopathy: A Report of the American Heart Association/American College of Cardiology Joint Committee on Clinical Practice Guidelines	Ommen SR	CIRCULATION	2024	10.1161/CIR.0000000000001250	256
Evaluation of Mavacamten in Symptomatic Patients With Nonobstructive Hypertrophic Cardiomyopathy	Ho CY	JACC	2020	10.1016/j.jacc.2020.03.064	229
Myosin Inhibition in Patients With Obstructive Hypertrophic Cardiomyopathy Referred for Septal Reduction Therapy	Desai MY	JACC	2022	10.1016/j.jacc.2022.04.048	221
Mavacamten Treatment for Obstructive Hypertrophic Cardiomyopathy: A Clinical Trial	Heitner SB	ANN INTERN MED	2019	10.7326/M18-3016	218
A small-molecule modulator of cardiac myosin acts on multiple stages of the myosin chemomechanical cycle	Kawas RF	J BIOL CHEM	2017	10.1074/jbc.M117.776815	161
Mavacamten for treatment of symptomatic obstructive hypertrophic cardiomyopathy (EXPLORER-HCM): health status analysis of a randomized, double-blind, placebo-controlled, phase 3 trial	Spertus JA	LANCET	2021	10.1016/S0140-6736 (21)00763-7	149
Hypertrophic cardiomyopathy mutations in MYBPC3 dysregulate myosin	Toepfer CN	SCI TRANSL MED	2019	10.1126/scitranslmed.aat1199	141

## 4. Discussion

The development of cardiac myosin inhibitors represents a transformative advancement in the management of HCM, shifting the therapeutic focus from empirical symptom control to molecularly targeted intervention.^[28–30]^ As a disease spectrum with diverse genetic underpinnings, HCM has conventionally been treated with broad-acting agents such as beta-blockers and non-dihydropyridine calcium channel blockers, while they cannot correct the primary sarcomeric dysfunction driving disease pathogenesis.^[31,32]^ The advent of precision therapeutics – notably mavacamten and aficamten – has enabled selective modulation of myosin’s mechanochemical activity, attenuating pathological hypercontractility while restoring diastolic compliance.^[33,34]^ This paradigm transcends incremental innovation, instead establishing a new era of disease-specific pharmacotherapy grounded in the fundamental biophysics of cardiac contraction.^[12]^

The bibliometric analysis indicates exponential growth in research output in this field between 2016 and 2025. Annual publication numbers gradually rose from single digits in 2016 to over 50 by 2023. The observed increase in publication output coincides temporally with key translational milestones: the foundational mechanistic studies (2016–2018), the pivotal clinical trials such as EXPLORER-HCM (2019–2022), and the postapproval integration phase (2023–2025). The linear regression model (*R*^2^ = 0.9345, *P* < .001) confirms this upward trend as statistically robust, indicating a sustained and accelerating research interest in this field over the study period. Notably, the inflection point in 2022 coincides with the FDA approval of mavacamten, catalyzing both academic interest and clinical adoption.^[31,35]^ This temporal coincidence may reflect an association between regulatory milestones and research output, although causality cannot be inferred from bibliometric data.

Geographically, the United States dominates the research landscape, contributing 54.6% of all publications and 24.6 citations per article, reflecting not only volume but also impact. However, the distribution is skewed; while Italy and Germany are secondary contributors, their citation per article ratios (33.8 and 2.1, respectively) reveal disparities in research influence. China, despite substantial output (23 articles), exhibits a low citation impact (4.7 per article), indicating a potential gap in translational relevance or international visibility. The co-authorship network further illustrates this imbalance: U.S. institutions form the central hub, with dense collaborations extending to Europe and, to a lesser extent, Asia. This asymmetry underscores the need for equitable global participation to ensure that therapeutic advances are generalizable across diverse genetic and clinical phenotypes.

Institutional productivity is concentrated among elite academic medical centers and pharmaceutical entities. Univ Penn leads with 39 publications and 1585 citations, followed by Bristol Myers Squibb (32 publications) and Brigham & Women’s Hospital (29 publications, 2085 citations). These institutions are prominent contributors to research output and citation impact in this field. The network analysis reveals collaborative patterns between academic medical centers and pharmaceutical companies, which may facilitate the translation of research findings into clinical applications. The network density (0.87) and modularity (0.62) metrics reveal tightly knit clusters with clear specialization: clinical research hubs (Mayo Clinic), translational science groups (University of Michigan), and international collaborations (Hannover Medical School). This collaborative architecture shows dense connections among a limited set of institutions, which may indicate potential barriers to broader global participation.

Keyword analysis delineates the field’s thematic evolution. Terms such as “mavacamten,” “left ventricular outflow obstruction,” and “myectomy” dominate the lexicon, reflecting the centrality of pharmacological and surgical interventions. The co-occurrence network identifies 3 interconnected clusters: pharmacological studies (centered on mavacamten), surgical interventions (myectomy), and diagnostic/prognostic markers (genetic analysis, N-terminal pro-B-type natriuretic peptide). The temporal shift from basic science (2016–2018) to clinical translation (2019–2025) is evident in the declining prominence of mechanistic keywords like “ATP turnover” and the rise of outcome-focused terms such as “KCCQ-12” and “symptomatic obstructive HCM.” This transition signifies the field’s successful navigation from molecular insight to patient-centered efficacy.

During the foundational period of 2016 to 2018, research predominantly focused on elucidating the fundamental molecular mechanisms underlying HCM pathophysiology. The keyword analysis reveals that basic science investigations concentrated on several key molecular targets: sarcomeric protein dysfunction: keywords such as “myosin heavy chain,” “troponin,” and “MYBPC3” dominated, reflecting intensive study of the sarcomeric apparatus as the primary pathogenic substrate. ATP hydrolysis and energy metabolism: Terms like “ATP turnover,” “myosin ATPase activity,” and “energy transduction” were prominent, indicating focus on the bioenergetic aspects of cardiac myosin function and the discovery that altered ATP utilization contributes to hypercontractility. Calcium handling mechanisms: keywords including “calcium sensitivity,” “troponin–tropomyosin complex,” and “excitation–contraction coupling” highlighted investigations into calcium-mediated regulation of contractile proteins. Protein folding and quality control: Emerging terms such as “protein aggregation,” “chaperone proteins,” and “unfolded protein response” reflected growing understanding of how mutant sarcomeric proteins trigger cellular stress pathways.

The transition to clinical translation after 2019 marked a paradigmatic shift toward patient-centered research themes: clinical efficacy endpoints: the emergence of keywords like “KCCQ-12,” “6-minute walk test,” and “peak oxygen consumption” reflected the field’s evolution toward standardized outcome measures for therapeutic interventions. Drug development and pharmacology: terms such as “mavacamten,” “aficamten,” “pharmacokinetics,” and “dose optimization” became central, indicating successful translation of molecular targets into therapeutic agents. Precision medicine approaches: keywords including “genotype–phenotype correlation,” “risk stratification,” and “personalized therapy” demonstrated the field’s progression toward individualized treatment strategies. Real-world evidence: the appearance of terms like “long-term safety,” “quality of life,” and “healthcare utilization” highlighted the expanding focus on postapproval surveillance and patient-reported outcomes.

Citation analysis reveals the intellectual scaffolding of the domain. The EXPLORER-HCM trial (729 citations) anchors the clinical evidence base, while foundational studies by Green et al (500 citations) and Anderson et al (274 citations) provide mechanistic underpinnings. Burst detection identifies 2016 to 2018 as the “basic science surge,” followed by a 2019 to 2021 “clinical validation phase,” and a 2022 to 2024 “guideline integration phase.” Notably, the 2024 AHA/ACC guidelines (256 citations) mark the crystallization of evidence into practice standards. This citation trajectory illustrates the chronological ordering of publications, proceeding from mechanistic studies to clinical validation and then to guideline-related works.

The publication and citation patterns observed in this analysis suggest that multiple factors may have contributed to the rapid progression of myosin inhibitor research from basic science to clinical application. The citation analysis reveals that the persistent clinical challenges in HCM management served as the primary catalyst for translational research. Traditional therapies (beta-blockers, calcium channel blockers) provided only symptomatic relief without addressing the underlying pathophysiology, leaving patients with limited treatment options. The high citation rates of early clinical studies (e.g., Heitner et al, 218 citations) reflect the medical community’s urgent need for disease-modifying therapies. The foundational work by Green et al (500 citations) provided a well-defined therapeutic target – cardiac myosin ATPase – with established pathophysiological relevance. Unlike many cardiovascular conditions where molecular mechanisms remain unclear, HCM research benefited from decades of genetic studies that had already identified sarcomeric proteins as disease drivers. This mechanistic clarity accelerated drug development by providing rational design targets. The citation network demonstrates convergence of multiple technological advances: advanced structural biology enabling myosin–drug interaction modeling, improved cardiac imaging allowing precise phenotyping, and genomic sequencing facilitating patient stratification.^[36–39]^ This technological synergy created unprecedented opportunities for precision therapeutic development.

The prominence of Bristol Myers Squibb (32 publications) and MyoKardia Inc. (22 publications) in the institutional analysis reflects significant private sector investment driven by the large patient population (estimated 1 in 500 individuals globally) and potential market size. The acquisition of MyoKardia by Bristol Myers Squibb for $13.1 billion in 2020 demonstrates the commercial validation of this therapeutic approach. The network analysis reveals robust collaborations between academic centers (Univ. Penn, Brigham & Women’s Hospital) and pharmaceutical companies, creating integrated research ecosystems that accelerated translation. These partnerships combined academic expertise in disease mechanisms with industry capabilities in drug development and clinical trial execution. The FDA’s breakthrough therapy designation for mavacamten in 2017 created regulatory incentives that accelerated development timelines and reduced financial risks for investors, catalyzing increased research investment.

The citation burst analysis shows that Anderson et al study (274 citations) provided crucial mechanistic validation by demonstrating how mavacamten stabilizes the super-relaxed state of myosin. This molecular-level proof-of-concept gave confidence to clinical investigators and regulatory agencies that the therapeutic approach was scientifically sound. The growing awareness of HCM through patient advocacy organizations and increased genetic testing created clinical demand for novel therapies. The citation patterns show increased focus on patient-reported outcomes (KCCQ-12, quality of life measures), reflectingthis patient-centered research momentum. The establishment of specialized HCM centers and clinical trial networks created the infrastructure necessary for rapid patient recruitment and standardized outcome assessment, enabling the successful execution of pivotal trials like EXPLORER-HCM.

The observed publication and citation patterns describe a temporal sequence from mechanistic studies to clinical trials and guideline publications. However, bibliometric data alone cannot identify the causal drivers of this pattern. The combination of a clear unmet clinical need, well-defined molecular targets, robust industry investment, a supportive regulatory environment, and established clinical infrastructure created a “perfect storm” for therapeutic development. The citation network demonstrates how each factor amplified the others: mechanistic clarity attracted industry investment, which enabled large-scale clinical trials, which generated regulatory approval, which stimulated further research investment, creating a self-reinforcing cycle of translational progress. These observed patterns may offer insights that could inform research strategies in other genetic cardiovascular diseases, though such extrapolations require further investigation.

The journal ecosystem further reinforces this translational narrative. The JACC (16 publications, 937 citations) and Circulation (6 publications, 378 citations) serve as premier dissemination venues, ensuring rapid uptake of high-impact findings. Mid-tier journals like Frontiers in Cardiovascular Medicine and JACC: Heart Failure provide platforms for specialized discourse, while the Sankey diagram illustrates robust cross-pollination between journals and research themes. This stratified dissemination model balances prestige with accessibility, critical for sustaining interdisciplinary engagement.

Safety-related terms, including “left ventricular ejection fraction reduction” and “risk evaluation and mitigation strategy,” appeared repeatedly in the keyword co-occurrence network, indicating that safety monitoring is a recurrent topic in the published literature. Keywords related to nonobstructive HCM also emerged in more recent publications, suggesting this remains an active area of investigation.

## 5. Limitations

This study presents a systematic bibliometric analysis of myosin inhibitor-targeted therapy for HCM, utilizing VOSviewer, CiteSpace, and the R package bibliometrix. The combined application of multiple analytical tools enhanced the objectivity and accuracy of the findings while also providing a macro-level perspective on the evolution of research topics and trends in this field. However, the study has the following limitations. First, the data were sourced exclusively from the WoSCC, and publications from other major databases such as Scopus and PubMed were not included. While the WoSCC is widely regarded as a high-quality data source for bibliometric analysis, its coverage is not exhaustive. This may result in incomplete coverage of the relevant literature, particularly regarding publications from regional journals or non-English sources, and affect the representativeness of the analysis. Publications indexed only in Scopus, PubMed, or regional databases may have been missed, and the geographic and institutional distributions reported here may partially reflect the coverage bias of the WoSCC rather than the true global research landscape. Future studies incorporating multi-databasetriangulation (e.g., WoS + Scopus + PubMed) would be needed to validate the patterns described in this analysis. Second, only English-language articles and reviews were included, which may lead to the omission of high-qualityresearchpublished in other languages and introduce language bias. This is particularly relevant for fields with significant research activity in non-English-speaking countries. Third, inconsistencies in author affiliation data over time (e.g., institutional name changes, mergers, or author mobility) could affect the accuracy of related statistical analyses, such as assessments of institutional productivity and collaboration networks. Fourth, bibliometric analysis by nature emphasizes quantitative metrics such as publication counts and citation frequencies, which may not fully capture the clinical significance or scientific quality of individual studies. Citation counts are also influenced by factors such as publication age and field-specific citation practices.

## 6. Conclusion

This bibliometric study, based on the WoSCC data, provides a descriptive overview of publication patterns as indexed in the WoSCC in myosin inhibitor research for HCM. The findings describe a field that has experienced substantial growth in publication output, with concentrated research activity in North America and Europe and collaborative networks centered on a limited number of institutions. The observed concentration of research activity in the United States and other high-income countries suggests a need for greater global diversification to ensure broader representation and generalizability of research findings. The collaborative patterns observed between academic institutions and industry partners, along with the prominent role of high-impact journals in disseminating research findings, characterize the current research landscape. However, the geographic concentration of research activity and the language restrictions of the current analysis highlight potential gaps that warrant attention in future studies.

Future directions should embrace real-world evidence, AI-enhanced monitoring, and combination strategies to characterize research trends related to myosin inhibitors in HCM.

## Acknowledgments

This work was supported by the Young Scientists Fund of the National Natural Science Foundation of China (Grant No. 82104677) and the Outstanding Young Scientific Talent Program of the China Academy of Chinese Medical Sciences (ZZ15-YQ-009). No individuals are named in this section. Correspondence concerning this article should be addressed to Qu Zhengyan (marked with an asterisk). Dr Qinghua Shang also acts as the co-corresponding author, jointly supervising this research and sharing responsibilities for manuscript revision and communications with the editorial office.

## Author contributions

**Data curation:** Lulu Yang, Xianbo Song, Haoran Fu, Heng Zhang.

**Funding acquisition:** Qinghua Shang.

**Methodology:** Ning Liu, Xianbo Song, Zhiyang Zhu, Zhengyan Qu.

**Software:** Jianming Wang, Zhiyang Zhu.

**Supervision:** Wende Tian, Jianming Wang, Zhengyan Qu, Qinghua Shang.

**Writing – original draft:** Ning Liu, Lulu Yang.

**Writing – review & editing:** Lulu Yang, Wende Tian, Zhengyan Qu, Qinghua Shang.
